# Suppression of Melatonin 2-Hydroxylase Increases Melatonin Production Leading to the Enhanced Abiotic Stress Tolerance against Cadmium, Senescence, Salt, and Tunicamycin in Rice Plants

**DOI:** 10.3390/biom9100589

**Published:** 2019-10-08

**Authors:** Geun-Hee Choi, Kyoungwhan Back

**Affiliations:** 1 Nakdonggang National Institute of Biological Resources, 137, Donam 2-gil, Sangju-si, Gyeongsangbuk-do 37242, Korea; ghchoi@nnibr.re.kr; 2 Department of Biotechnology, Chonnam National University, Gwangju 61186, Korea

**Keywords:** cadmium, diurnal rhythm, endoplasmic reticulum stress, salt, senescence, transgenic rice

## Abstract

Melatonin 2-hydroxylase (M2H) catalyzes the conversion of melatonin into 2-hydroxymelatonin (2OHM), which is present in plants at a higher concentration than melatonin. Although *M2H* has been cloned, the in vivo function of its product is unknown. Here, we generated stable T_2_ homozygous transgenic rice plants in which expression of endogenous *M2H* was suppressed (RNAi lines). However, we failed to generate *M2H* overexpression transgenic rice due to failure of somatic embryogenesis. The *M2H* transcript level showed a diurnal rhythm with a peak at night concomitantly with the peak concentration of 2OHM. RNAi rice showed a reduced *M2H* mRNA level and 2OHM and melatonin concentrations. The unexpected decrease in the melatonin concentration was caused by redirection of melatonin into cyclic 3-hydroxymelatonin via a detour catabolic pathway. Thus, the decrease in the melatonin concentration in *M2H* RNAi rice led to slowed seedling growth and delayed germination. By contrast, the transient increase in the melatonin concentration was of greater magnitude in the *M2H* RNAi than the wild-type rice upon cadmium treatment due to possible suppression of melatonin degradation. Due to its higher concentration of melatonin, the *M2H* RNAi rice displayed tolerance to senescence, salt, and tunicamycin stresses. Therefore, the increase in the melatonin concentration caused by suppression of melatonin degradation or by overexpression of melatonin biosynthetic genes enhances stress tolerance in rice.

## 1. Introduction

The emergence of melatonin on Earth is estimated to have occurred several billion years ago, and to be closely synchronized with the appearance of non-purple sulfur bacteria and prokaryotic cyanobacteria [1]. Due to their possible evolution into mitochondria and chloroplasts, respectively, all living cells harboring these organelles are proposed to synthesize melatonin. Despite being such an ancient molecule, the initial function of melatonin as a potent antioxidant is well conserved in all currently living organisms, including bacteria, animals, and plants [1,2]. Further complex and pleiotropic functions of melatonin in animals and plants have been acquired independently during the diversification of organismal evolution [1]. For example, melatonin is a hormone regulating the circadian rhythm and sleep regulation in animals by involvement of multiple melatonin receptors [3]. Similar to the diverse array of functions in animals, plants have also been found to exhibit a large number of physiological functions as a bio-stimulator or hormone for plant growth and development, as well as a signaling molecule conferring tolerance against a wide range of biotic and abiotic stresses [4]. In more detail, despite the short history of plant melatonin research compared to that in animals [5,6,7], it was clearly documented that melatonin plays diverse roles, including in growth and development [8,9,10,11,12], defense against pathogens [13,14,15,16], and tolerance to a number of abiotic stresses such as cold [17], drought [18], senescence [19], salt [20], heat [21], endoplasmic reticulum (ER) stress [22], high light [23], and oxidation [18,24,25].

**Figure 1:**
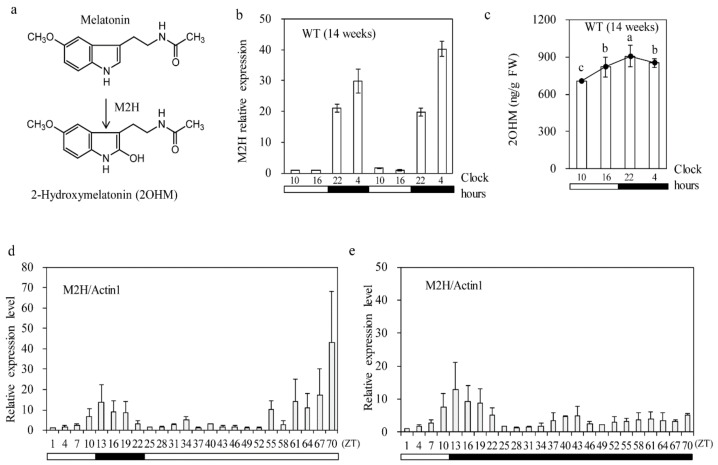
Diurnal expression of rice melatonin 2-hydroxylase (*M2H*) and 2OHM. (**a**) Enzymatic reaction of M2H. (**b**) *M2H* mRNA level in the leaves of 14-week-old rice grown in the field (darkness from 20:00 to 06:30 h). (**c**) 2OHM level during the light/dark cycle in 14-week-old rice. (**d**) Diurnal expression of *M2H* grown under a 12 h light/dark cycle, followed by 46 h of continuous light. (**e**) Diurnal expression of *M2H* grown under a 12 h light/dark cycle, followed by 46 h of continuous darkness. Fourteen-day-old rice seedlings were employed (**d**,**e**), and total RNA samples were collected at 3 h intervals from the aerial parts of the plants. Horizontal bars below indicate light (white) and dark (black) conditions. Values are means ± standard deviations of three independent experiments. FW, fresh weight. ZT, Zeitgeber time (ZT 0, beginning of light period).

In addition to the successful cloning of all genes involved in melatonin biosynthesis from tryptophan in plants [26], the catabolic pathway of melatonin has also been relatively well uncovered in recent years [7]. In rice seedlings, it was reported that exogenous melatonin application led to the production of several melatonin metabolites, including 2-hydroxymelatonin (2OHM), cyclic 3-hydroxymelatonin (c3OHM), N^1^-acetyl-N^2^-formyl-5-methoxykynuramine (AFMK), and N^2^-acetyl-5-methoxykynuramine (AMK), suggestive of the presence of a melatonin metabolism pathway [27]. In marked contrast to animals, which produce 2OHM by the oxidation of melatonin in response to ultraviolet (UV) and chemical treatments [28,29] via non-enzymatic reactions, plants are capable of synthesizing 2OHM by the action of melatonin 2-hydroxylase (M2H) enzyme (Figure 1A) [30]. Of note, the levels of 2OHM are considerably higher than those of melatonin in various plant species [31], suggestive of a certain physiological role for 2OHM in plants. In terms of the chemical structure of 2OHM, a tautomer of 2OHM, named 2-oxomelatonin, was already detected in chromatograms containing the 2-hydroxylated melatonin metabolite in vitro [7], however, the detection of 2OHM as a plant natural product has not been reported thus far despite the structural identification of a huge variety of plant secondary metabolites [32]. Thus, it would be interesting to determine the function of 2OHM as a novel metabolite in plants.

We previously reported that 2OHM induces mitogen-activated protein (MAP) kinase activation comparable to that by melatonin in *Arabidopsis* [33]. In support of a functional role for 2OHM, exogenous 2OHM treatment enhances tolerance to combined cold and drought stress in several plant species including rice and tobacco [34,35]. However, no genetic or molecular functional analyses of 2OHM have been undertaken. To unveil the roles of 2OHM in plants, we characterized not only its diurnal expression patterns in view of mRNA and 2OHM levels but also generated transgenic rice plants which overexpressed and suppressed the *M2H* gene to determine whether over- or under-production of 2OHM plays a role in plant growth, development, and response to abiotic stresses.

## 2. Materials and Methods

### 2.1. Generation of Transgenic Rice Plants Over- and Under-Expressing M2H

To over-express rice *M2H* (GenBank accession number AK119413), full-length *M2H* was introduced into the pIPKb002 binary vector as described previously [19]. In brief, full-length rice *M2H* cDNA was first amplified by polymerase chain reaction (PCR) using a primer set (forward primer 5’-AAA AAG CAG GCT CCA TGC CCG CCG TGG CCG-3’; reverse primer 5’-AGA AAG CTG GGT TCA GGG TTT GTC GAT-3’) and a cDNA provided by the National Institute of Agrobiological Sciences [36]. The PCR product was further amplified using the *att*B adaptor primer set (forward primer, 5´-GGG GAC AAG TTT GTA CAA AAA AGC AGG CT-3´; reverse primer, 5´-GGG GAC CAC TTT GTA CAA GAA AGC TGG GT-3´). The resulting rice *M2H* PCR product was gel-purified and cloned into the pDONR221 Gateway vector (Invitrogen, Carlsbad, CA, USA) via the BP (*att*B × *att*P) recombination reaction. Next, the pDONR221-M2H entry vector was recombined with the pIPKb002 destination vector via LR (*att*L × *att*R) recombination to yield pIPKb002-M2H, which was transformed into *Agrobacterium tumefaciens* strain LBA4404. To suppress *M2H* expression, the pTCK303 binary vector was used as previously described [37]. Briefly, an N-terminal 325 bp *M2H* cDNA fragment was amplified by PCR using the following primer set: *M2*H-F 5’-ACT AGT ATG CCC GCCGTG GCC-3’ (*Spe*I site underlined) and *M2H*-R 5’-GAG CTC GTG TCG TACCTG-3’ (*Sac*I site underlined) and the *M2H* cDNA. The *M2H* PCR product was first cloned into the T&A cloning vector (T&A:M2H; RBC Bioscience, New Taipei City, Taiwan) for further cloning experiments. From the T&A:M2H plasmid, the antisense *M2H* insert was digested by *Sac*I and *Spe*I, while the sense *M2H* insert was digested by *Kpn*I and *Bam*HI in the T&A vector. The antisense *M2H* fragment (*Sa*cI and *Sp*eI insert) was first ligated into the pTCK303 vector. Thereafter, the sense fragment of the *M2H* insert was sequentially ligated into the pTCK303 vector plasmid harboring the corresponding *M2H* antisense fragment. The resulting pTCK303:M2H RNAi binary vector was transformed into *A. tumefaciens* LBA4404, and into rice (*Oryza sativa* Dongjin) as previously described [10].

### 2.2. Plant Growing Conditions

The dehusked rice seeds were surface-sterilized and grown on half-strength Murashige and Skoog (MS) medium (MB Cell, Seoul, Korea) in vertically oriented polystyrene square dishes (SPL Life Science, Pocheon-si, Korea). The rice seedlings were grown at 28 °C under a 12/12 h light/dark cycle at a photosynthetic photon flux density of 100 µmol/m^2^/s^1^ for seven days. In addition, rice seeds were soaked in distilled water for three days at 28 °C, and the germinated seeds were transplanted into soil under the conditions described above.

### 2.3. Semi-Quantitative and Quantitative Real-Time Reverse Transcription Polymerase Chain Reaction 

The RNeasy Plant Mini Kit (Qiagen, Tokyo, Japan) was used to extract total RNA from the rice samples, which was subjected to DNase I (Qiagen) treatment to remove any contaminating DNA. Aliquots (1 µg) of total RNA were reverse-transcribed using RevertAid reverse transcriptase (Thermo Scientific Fermentas, St. Leon-Rot, Germany) with 500 ng of oligo(dT)18 primer at 42 °C for 1 h. Aliquots (0.2 µL) of cDNA were used as templates for PCR amplification with the appropriate primer sets using rice UBQ5 as an equal loading control as previously described [38]. Real-time PCR was performed on a Mic qPCR Cycler system (Bio Molecular Systems, Queensland, Australia) as previously described [10,19] using specific primers for *ATPase a*, *HKT1*, *P5CR*, *rbcL*, and *sodCp* [34]. Other primer set for various genes were follows: *M2H* (forward 5′-TGC TTT GAG GTG GTG AAC C-3′; reverse 5′-ATC AGA GTG AGG AGC CAA GC-3′), *Actin*1 (forward 5′-TGC TAT GTA CGT CGC CAT CCA G-3′; reverse 5′-AAT GAG TAA CCA CGC TCC GTC A-3′), *M3H* (forward 5′-CCA CTA GTA TGG CGG GAG CAA GAT-3′; reverse 5′-ATG AGC TCC CAG AAC CTT AGG TCC-3′), *BIP1* (forward 5′-TGA ACG TGA AGG CTG AGG AC-3′; reverse 5′-GTA GGT CTC GAG CTG GTT GC-3′), *BIP4* (forward 5′-CAA GGA GGA GTA CGA GGA GAA G-3′; reverse 5′-CAC ACT TTC GAT CGA ATC CAA AC-3′), *CNX* (forward 5′-CGC CGG AGG TCC CGA AGG GAG ACA-3′; reverse 5′-CTA GCT TGC ACT GAA CCT CAC AC-3′), and *PDIL1-*1 (forward 5′-ACA AGT GAG TAG GAG AGG GCA TGG-3′; reverse 5′- CAA CTT GTC CAG AAC CAC TTC TCT A-3′). GenBank accession numbers were *M2H* (AK119413), *M3H* (AK067086), *Actin*1 (Os03g50885), *UBQ*5 (AK061988), *ATPase a* (AK121120), *rbcL* (KM088016), *HKT1* (AB061311), *sodC*p (AB026724), *P5C*R (AK070184), *BIP*1 (AK119653), *BIP4* (AK106696), *CN*K (AK069118), and *PDIL1*-1 (AK068268).

### 2.4. Malondialdehyde, Hydrogen Peroxide, and Electrolyte Leakage Assays

To measure malondialdehyde (MDA) concentrations, frozen rice tissues (50 mg) were ground to a powder in liquid nitrogen using a TissueLyser II (Qiagen) and extracted with 1.5 mL homogenization buffer containing 0.5% thiobarbituric acid (TBA) and 20% trichloroacetic acid (TCA). The supernatant was centrifuged at 13,000× *g* for 15 min, boiled at 95 °C for 30 min, and placed on ice for 5 min. The MDA concentration was determined at wavelengths of 440, 532, and 600 nm using a spectrophotometer (Optizen Pop-Bio). Malondialdehyde (MDA) was quantified using a molar extinction coefficient of 156/nmol/L/cm as previously described [39]. To measure electrolyte leakage, detached leaves, leaf disks, or seedlings were challenged with various stresses. The samples were placed in 50 mL tubes containing 15 mL distilled H_2_O or 3 mM MES (pH 5.8) buffer and the conductivity of the solution was measured using a conductivity meter (Cole-Parmer Instrument Co., Vernon Hills, IL, USA). To measure the hydrogen peroxide concentration, frozen leaves (0.05 g) were ground to a powder under liquid nitrogen and homogenized with 1 mL homogenization buffer (50 mM Na-P (pH 6.5), 1 mM hydroxylamine). The extract was centrifuged at 12,500× *g* for 15 min at room temperature. The supernatant was collected and mixed with 0.5 mL reaction buffer (20% H_2_SO_4_, 0.1% titanium chloride). The extract was clarified by centrifugation at 12,500× *g* for 10 min. The H_2_O_2_ concentration was determined at 410 nm using a spectrophotometer (Optizen, Seoul, Korea). H_2_O_2_ was quantified using a molar extinction coefficient of 0.28/μmol/L/cm.

### 2.5. Measurement of 2OHM, c3OHM, and Melatonin Concentrations

Melatonin and 3-hydroxymelatonin concentrations were quantified by high-performance liquid chromatography (HPLC) as previously described [27]. Pulverized rice samples (0.1 g) were extracted with 1 mL chloroform for 1 h at room temperature. The chloroform extracts (200 µL) were completely evaporated and dissolved in 0.1 mL 40% methanol, and 10 µL aliquots were subjected to HPLC with fluorescence detection (Waters, Milford, MA, USA) using excitation at 280 nm and emission at 348 nm. For quantification of the 2OHM concentration, the same 10 µL aliquots were subjected to HPLC with a UV detector (Waters) at 254 nm. The 2OHM was separated on a Sunfire C18 column (Waters; 4.6 × 150 mm) using isocratic elution with 35% MeOH in 0.3% TFA for 25 min at a flow rate of 0.25 mL/min; 2OHM was eluted at around 18.9 min. All measurements were performed in triplicate.

### 2.6. Abiotic Stress Treatments

Senescence treatments were performed in two different ways. First, 10 detached leaves of rice plants grown for six weeks in soil pots were incubated in 50 mL polypropylene conical tubes containing water and subsequently at 25 °C for seven days under a 12/12 h light/dark cycle. Second, rice leaves were cut into 1 cm diameter disks and floated on 3 mM MES (pH 5.8) buffer at 25 °C under dark conditions for five days. For cadmium treatment, a group of 10 segments (detached leaves from 6-week-old plants grown in a soil pot) were transferred to 50 mL polypropylene conical tubes containing 25 mL water with or without 0.2 mM CdCl_2_ and incubated for the indicated times under a 12/12 h light/dark cycle. For tunicamycin (Tm) treatment, 8-day-old rice seedlings grown in half-strength MS medium were challenged with 1 µg/mL Tm for two days under a 12/12 h light/dark cycle at 25 °C. Next, Tm-treated seedlings were transferred to conical tubes without Tm for two days for recovery. Salt treatment was carried out using 2-week-old rice plants grown in soil pots immersed in 0.2 M NaCl for three days under a 12/12 h light/dark cycle at 25 °C. All rice tissues were immediately frozen in liquid nitrogen and stored at −80 °C for further analyses.

### 2.7. Chlorophyll Concentration Assay

Rice samples stored at −80 °C were pulverized to powder using a TissueLyser II instrument (Qiagen, Tokyo, Japan). The powder (100 mg) was extracted with 1 mL 0.1 M NH_4_OH (containing 80% acetone). Chlorophyll concentrations were determined at wavelengths of 647, 644, and 750 nm using a spectrophotometer (Optizen POP- Bio; Mecasys, Daejeon, Korea) as previously described [40].

### 2.8. Germination Test

For germination tests, healthy and uniform seeds were surface-sterilized with 100% ethanol for 30 s followed by 2% sodium hypochlorite for 5 min. The resulting seeds were rinsed three times in deionized water for 5 min. Twenty-five seeds were placed in 3 mL distilled water in 6-well plates, which were wrapped in transparent plastic to prevent water evaporation. The seeds were germinated at 28 °C under LD (14 h light/10 h dark) conditions. The germination percentage was calculated after 36 h. Seeds were considered to have germinated if the length of the emerging shoot was >1 mm. Each treatment comprised 3 replicates, each of 25 seeds.

### 2.9. Statistical Analysis

Data were subjected to analysis of variance using IBM SPSS Statistics 23 software (IBM Corp. Armonk, NY, USA). Means with different letters or asterisks are significantly different at *p* < 0.05 according to Tukey’s post hoc honestly significant difference (HSD) test. Data are presented as means ± standard deviations of triplicate determinations.

## 3. Results

### 3.1. Diurnal M2H mRNA Level Rhythm

According to the Rice XPro Database [41], *M2H* expression in rice peaks at night during the entire growth stage; in contrast, *M3H* expression peaks at night during late vegetative growth peaks around eight weeks after seeding [42]. To confirm the diurnal rhythm of *M2H* expression, we quantified the *M2H* mRNA level in 14-week-old rice leaves growing in the field by qRT-PCR. As shown in Figure 1b the *M2H* mRNA level was minimal during the day and increased > 25-fold at night, suggestive of a diurnal rhythm. Similarly, the 2OHM level also peaked at night (*M2H* mRNA level in 14-week-old rice leaves growing in the field by qRT-PCR. As shown in Figure 1C). To assess whether this diurnal *M2H* expression was operated by a circadian rhythm, 2-week-old rice seedlings were subjected to a 12/12 h light/dark cycle followed by 48 h of continuous light (LL) or darkness (DD). Diurnal *M2H* expression was not observed under the LL or DD condition, indicative of a dark-induced diurnal rhythm rather than a free running internal circadian rhythm. Notably, *M2H* expression significantly increased at the late stage of the LL condition, indicating that *M2H* responds to continuous light stress as does *DWARF4*, a key brassinosteroid biosynthesis gene [10]. Conversely, *M3H* expression was increased significantly under the DD condition, despite its similar diurnal rhythm to *M2H* [42]. This finding suggests that *M2H* and *M3H* have different functions in rice plants.

### 3.2. Rice Shoot Regeneration from Calli by Somatic Embryogenesis

**Figure 2:**
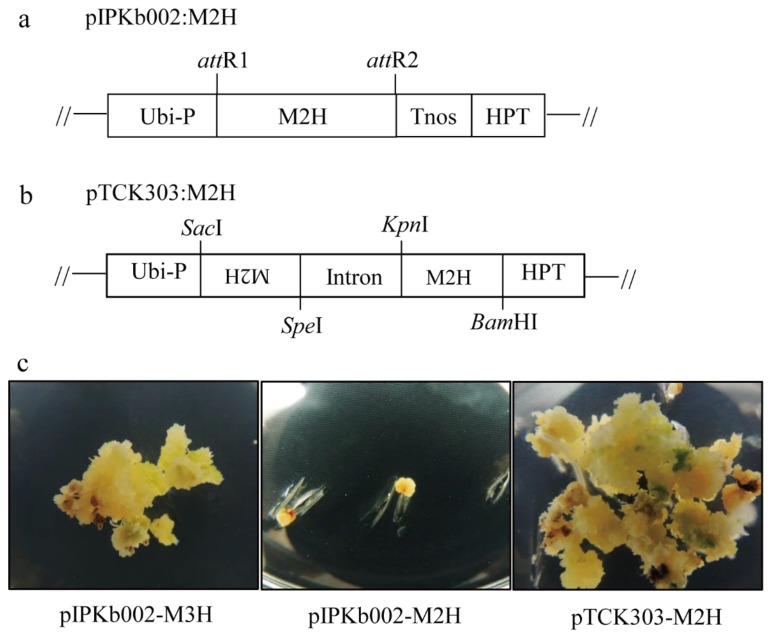
Binary vector structures and rice somatic embryogenesis. (**a**) Schematic diagram of the overexpression binary vector (pIPKb002:M2H). (**b**) RNAi binary vector (pTCK303:M2H). (**c**) Rice somatic embryogenesis from calli. *M2H*, *Oryza sativa* melatonin 2-hydroxylase; *M3H*, *Oryza sativa* melatonin 3-hydroxylase; *Ubi-P*, maize ubiquitin promoter; *HPT*, hygromycin phosphotransferase; *Tnos*, nopaline synthase terminator; attR1 and attR2, recombination sites; WT, wild type. Rice calli were transferred to N6 regeneration medium containing 1-napthylacetic acid 0.5 mg/L, 6-benzylaminopurine 3 mg/L, and 50 mg/L hygromycin for somatic embryogenesis.

To generate transgenic rice plants, scutellum-derived calli were co-cultured with *Agrobacterium* harboring the binary vector for three days, during which T-DNA was incorporated into the rice genome. Thereafter, calli were screened on N6 selection medium containing 2 mg/L 2,4-D and 50 mg/L hygromycin for 30 days in the dark to generate transgenic calli expressing the transgene. All three transgenes were successfully transformed into the rice genome, as indicated by generation of healthy hygromycin-resistant calli. *M3H*, which is responsible for cyclic 3-hydroxymelatonin (c3OHM) synthesis, was employed as a transformation control because it is readily transformed and regenerated via somatic embryogenesis [42]. However, when the three transgenic calli were transferred to regeneration medium under a 12/12 h light/dark cycle for shoot formation via somatic embryogenesis, the *M3H* overexpression and *M2H* downregulation calli (RNAi) grew rapidly and generated shoots (Figure 2c) whereas the *M2H* overexpression calli failed to grow or regenerate. These data suggested that the overproduction of 2OHM caused by overexpression of *M2H* was detrimental to calli growth and somatic embryogenesis under the light condition. The reason for this failure is not known, but it is possible that 2OHM production inhibits plant growth by reducing the melatonin level, which is implicated in shoot organogenesis [12]. Therefore, we generated only *M2H*-downregulated transgenic rice plants by RNAi.

### 3.3. Reduced 2OHM Production in M2H RNAi Transgenic Rice

**Figure 3:**
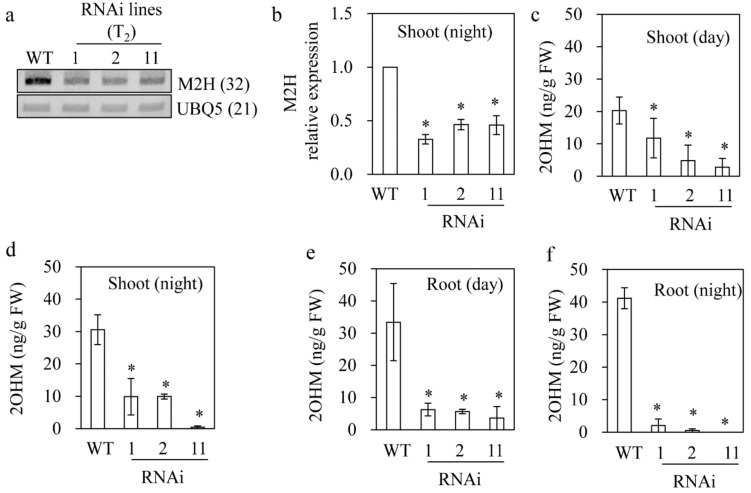
Molecular characterization of *M2H* RNAi transgenic rice. (**a**) RT-PCR analyses of *M2H* expression in shoot during the night. (**b**) qRT-PCR analyses of *M2H* expression in shoot during the night. (**c**) 2OHM content in shoot during the day. (**d**) 2OHM content in shoot during the night. (**e**) 2OHM content in root during the day. (**f**) 2OHM content in root during the night. T_2_ homozygous seedlings of the wild type (WT) and *M2H* RNAi rice grown in half-strength MS medium for seven days were dissected into shoot and root for analyses. FW, fresh weight. * Significant differences from the wild-type (*p* < 0.05). Numbers in parentheses are the numbers of PCR cycles.

After obtaining a total of 15 independent transgenic seeds (T_1_), we generated T_2_ homozygous rice plants in which *M2H* was downregulated by selfing and screening T_2_ seeds. Consequently, three independent T_2_ RNAi lines were chosen for examination of the phenotypic, biochemical, and molecular features. The *M2H* mRNA level was significantly downregulated in seven-day-old seedlings of all of the RNAi lines (Figure 3a). The *M2H* mRNA level was four-fold lower in the RNAi lines than the wild type (Figure 3b). Moreover, the level of 2OHM in the shoot and root of 7-day-old seedlings was lower in the *M2H* RNAi rice than in the wild type. The wild-type shoots produced 2OHM at 20 ng/g fresh weight (FW) during the day compared to 6 ng/g FW for the RNAi lines on average. During the night, the 2OHM level in shoot was 30 ng/g FW in the wild type, which was 1.5-fold higher than that of the wild type during the day (Figure 3c,d), indicative of night increase of 2OHM. This night increase on 2OHM was also observed in the root (Figure 3e,f). The root of the RNAi lines also had a lower level of 2OHM than that of the wild type (Figure 3e,f). Therefore, *M2H* expression is linked to 2OHM production in rice seedlings, suggesting that *M2H* is associated with 2OHM synthesis in plants.

### 3.4. Germination and Seedling Growth of M2H RNAi Rice

**Figure 4:**
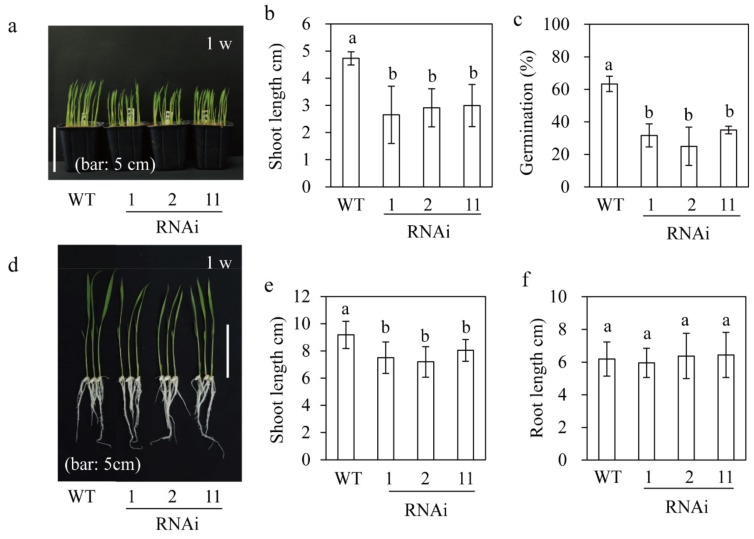
Morphology and germination rate of *M2H* RNAi rice. (**a**) Photograph of rice plants grown for one week in a soil pot. (**b**) Shoot length of one-week-old seedlings in a soil pot. (**c**) Germination rate. (**d**) Photograph of rice seedlings grown in half strength MS medium for one week. (**e**) Shoot length of seedlings grown in MS medium. (**f**) Root length of seedlings grown in MS medium. WT, wild type; R1-R11, *M2H* RNAi rice. Different letters denote significant differences as determined by Tukey’s post hoc honestly significant difference (HSD) test at *p* < 0.05. Germination was measured in three replications, each of 25 seeds.

Exogenous melatonin application promotes germination and seedling growth in cucumber and wheat [17,43]. However, no similar study or one involving transgenic plant over- or under-producing serotonin *N*-acetyltransferase (*SNAT1*), which encodes the penultimate step enzyme in melatonin biosynthesis [9,19,40], has been conducted in rice plants. Thus, we investigated seedling growth and germination of *M2H* RNAi rice. First, *M2H* RNAi seeds were seeded in soil pots and cultured in a growth chamber for seven days under a 12/12 h light/dark cycle at 28 °C. The *M2H* RNAi seedlings were shorter than the wild type (Figure 4a,b), indicative of involvement of 2OHM in growth. Next, *M2H* transgenic seeds were germinated in a Petri dish containing water under LD condition for 36 h. The germination rate of the wild type was 60%, compared to 30% for the RNAi lines, indicative of the inhibition of germination by *M2H* suppression. To evaluate root growth, dehusked seeds were surface-sterilized and seeded on half-strength MS medium at 28 °C for seven days under a 12/12 h light/dark cycle. The *M2H* RNAi lines showed reduced seedling growth as did in the soil pot (Figure 4d,e) whereas root growth was comparable to that of the wild type (Figure 4f). Unlike the semi-dwarf with erect leaf phenotype of the *SNAT2* and *COMT* RNAi lines [44], the *M2H* RNAi lines did not show an erect-leaf phenotype, suggestive of the irrelevance of brassinosteroid (BR) synthesis [10,44]. In contrast to the inhibition of shoot and root growth by *SNAT1* and *SNAT2* suppression [9,10], *M2H* suppression inhibited only shoot growth, indicative of a different physiological role for *M2H* compared to *SNAT1* and *SNAT2*.

### 3.5. Melatonin Reduction in the M2H RNAi Rice

**Figure 5:**
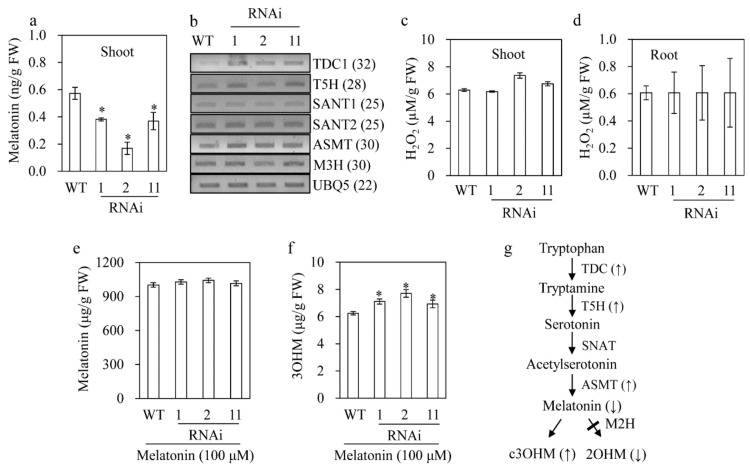
Melatonin production in the *M2H* RNAi rice seedlings. (**a**) Melatonin level. (**b**) RT-PCR analyses of the expression of melatonin biosynthetic genes. (**c**) Hydrogen peroxide level in shoot. (**d**) Hydrogen peroxide level in root. (**e**) Shoot melatonin content in seven-day-old seedlings pretreated with 100 μM melatonin for 24 h. (**f**) c3OHM content in seven-day-old seedlings pretreated with 100 μM melatonin for 24 h. (**g**) Proposed model of melatonin regulation by suppression of 2OHM. Rice seedlings grown for seven days in MS medium were treated with 100 μM melatonin for 24 h, followed by HPLC analyses. FW, fresh weight. * Significant differences as determined by Tukey’s post hoc HSD test at *p* < 0.05.

To examine whether the reduced seedling growth in the *M2H* RNAi rice was attributable solely to 2OHM deficiency, we measured the melatonin concentration. The *M2H* RNAi rice had a lower melatonin concentration than the wild type (Figure 5a). This suggested feedback regulation between melatonin and 2OHM synthesis. Therefore, we measured the transcript levels of the following genes related to melatonin biosynthesis: tryptophan decarboxylase (TDC), tryptamine 5-hydroxylase (T5H), SNAT, and *N*-acetylserotonin *O*-methyltransferase (ASMT). The transcript level of *TD*C, *T5H* and *ASMT* was elevated in the *M2H* RNAi rice compared to the wild type, indicating a reduced melatonin concentration in the *M2H* RNAi rice based on the feedback regulation of genes. To determine whether the reduced seedling growth in the *M2H* RNAi rice is associated with reactive oxygen species (ROS), we measured the level of H_2_O_2_. The H_2_O_2_ level did not differ between the wild-type and RNAi rice, suggesting that ROS are not involved in the reduced seedling growth of the latter.

To assess whether the inhibition of 2OHM synthesis by *M2H* suppression, rather than melatonin, affects the synthesis of c3OHM, we quantified the c3OHM concentration in seven-day-old seedlings treated with 100 µM melatonin rhizospherically for 24 h. As shown in Figure 5f, the c3OHM concentration was higher in the *M2H* RNAi lines compared to the wild type (Figure 5g), whereas the *M3H* mRNA levels were similar (Figure 5b). Due to the exogenous melatonin treatment, the mean melatonin concentration in the *M2H* RNAi lines was 1000 µg/g FW, comparable to that of the wild type (Figure 5e). Therefore, the reduced 2OHM concentration caused by *M2H* suppression facilitates melatonin conversion to c3OHM, resulting in a decrease in the melatonin concentration, suggestive of feedback regulation among melatonin, 2OHM, and c3OHM (Figure 5g).

### 3.6. M2H RNAi Rice Produced More Melatonin upon Cadmium Treatment Resulting in Tolerance against Cadmium Stress

**Figure 6:**
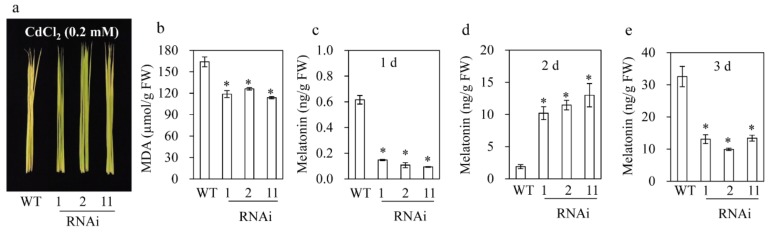
Increased transient melatonin production induces cadmium tolerance in the *M2H* RNAi rice. (**a**) Photograph after cadmium treatment (three days). (**b**) Malondialdehyde (MDA) level. (**c**) Melatonin level at one day after cadmium treatment. (**d**) Melatonin level at two days after cadmium treatment. (**e**) Melatonin level at three days after cadmium treatment. Six-week old rice leaves were detached and challenged with cadmium (0.2 mM) under a 12/12 h light/dark cycle at 28 °C. WT, wild type; FW, fresh weight. * Significant differences as determined by Tukey’s post hoc HSD test at *p* < 0.05.

In rice, an increased melatonin level due to overexpression of *SNAT1* conferred tolerance to cadmium stress [19]. To examine cadmium sensitivity, detached leaves of six-week-old *M2H* RNAi rice were challenged with 0.2 mM CdCl_2_ under a 12/12 h light/dark cycle and incubated for the indicated times. The *M2H* RNAi rice showed increased cadmium tolerance compared to the wild type, as indicated by a lower MDA content (Figure 6a,b). Moreover, one day after cadmium treatment, the melatonin level was lower in the *M2H* RNAi than the wild type as were shown in the untreated seedlings (Figure 6c). However, the melatonin level was five-fold higher in the *M2H* RNAi rice than in the wild type two days after treatment, suggesting that melatonin accumulates due to impaired 2OHM production in the presence of cadmium (Figure 6d). However, no further increase in the melatonin concentration occurred in the *M2H* RNAi lines after five days, whereas that in wild type increased to 32 ng/g FW (Figure 6e). These data indicate the functional importance in plants for melatonin metabolism and cadmium resistance of an early increase in, rather than the final level of, melatonin. The feedback regulation of melatonin production by *M2H* suppression in the normal growth stage is absent during the middle phase of melatonin induction such as cadmium treatment. Thus, cadmium tolerance in *M2H* RNAi rice is attributable to an increased melatonin concentration, as in *SNAT1* overexpression rice [19].

### 3.7. M2H RNAi Rice Showed Delayed Leaf Senescence

**Figure 7:**
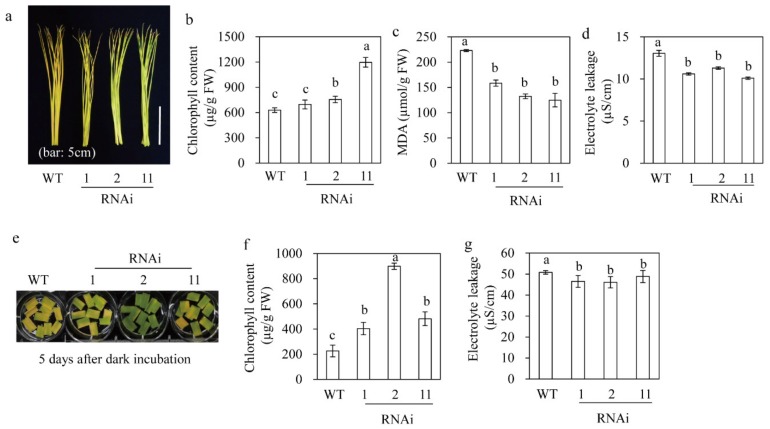
Senescence tolerance of the *M2H* RNAi rice. (**a**) Photograph after senescence treatment. (**b**) Chlorophyll level. (**c**) MDA level. (**d**) Electrolyte leakage level. (**e**) Photograph after leak disk senescence assay. (**f**) Chlorophyll level. (**g**) Electrolyte leakage level. Six-week-old rice leaves grown in a soil pot in a growth chamber were subjected to senescence treatments. (**a**–**d**) Detached leaves (about 15 cm length) were employed for seven days. (**e**–**g**) Leaf disks (about 1 cm diameter) were floated on 3 mM MES buffer (pH 5.8) and incubated in the dark for five days. WT, wild type; FW, fresh weight. Different letters denote significant differences as determined by Tukey’s post hoc HSD test at *p* < 0.05.

We subjected six-week-old rice leaves grown in soil pots to two different senescence assays. Detached rice leaves incubated in distilled water for seven days turned yellow due to chlorophyll degradation (Figure 7a). The *M2H* RNAi rice had a higher level of chlorophyll than the wild type, although that of RNAi line 1 was only marginally higher than that of the wild type (Figure 7b). The MDA concentration was 1.6-fold lower in the RNAi rice than in the wild type, suggesting resistance to senescence (Figure 7c). Similarly, electrolyte leakage was decreased in the RNAi rice relative to the wild type (Figure 7d). In analogy, leaf senescence was also delayed in the RNAi rice when analyzed by leaf disk assay. For example, the RNAi rice had higher chlorophyll and lower electrolyte leakage levels than the wild type (Figure 7d–f), consistent with those in detached leaves. Therefore, the increased melatonin concentration by *M2H* suppression enhances resistance to senescence.

### 3.8. M2H RNAi Rice Exhibited Resistance to Salt and Tunicamycin Stresses

**Figure 8:**
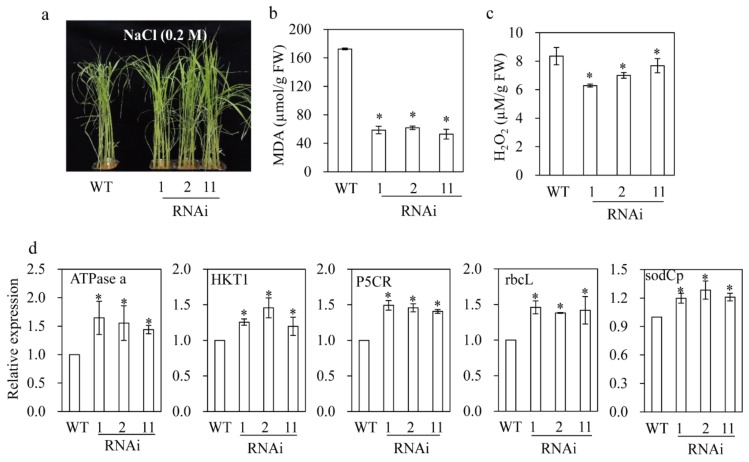
Salt tolerance in *M2H* RNAi rice. (**a**) Representative phenotypes of wild type and *M2H* RNAi rice in response to 0.2 M NaCl. (**b**) MDA level. (**c**) Hydrogen peroxide level. (**d**) Relative expression levels of various stress-related genes. Two-week-old rice seedlings were dipped into 0.2 M NaCl solution for three days under a 12 h light/dark cycle at 28 °C. GenBank accession numbers: *ATPase a* (AK12120), *HKT1* (AB061311), *P5C*R (AK070184), *rbcL* (KM088016), *sodCp* (AB026724).

Two-week-old rice seedlings grown in soil pots were challenged with 0.2 M NaCl for three days. The *M2H* RNAi rice appeared healthier than the wild type (Figure 8a); this was supported by the MDA and H_2_O_2_ concentrations. The MDA concentration was three-fold lower in the *M2H* RNAi rice than in the wild type (Figure 8b). In addition, the H_2_O_2_ concentration was lower in the *M2H* RNAi rice than in the wild type (Figure 8c). The molecular data confirmed the salt tolerance of the *M2H* RNAi rice. The expression of several stress marker genes was significantly elevated in the *M2H* RNAi rice compared to the wild type. For example, the expression levels of *ATPase a* (electron transport chain) and *rbcL* (photosynthesis) in the *M2H* RNAi rice were higher than those in the wild type (Figure 8d). The expression of pyrroline 5-carboxylate reductase (*P5CR*; proline biosynthesis) and superoxide dismutase (*sodCp*; ROS scavenging) was enhanced in the *M2H* RNAi rice. In particular, the expression of the potassium transporter gene *HKT1*, which inhibits the transport of Na^+^ to the shoot, was increased in the *M2H* RNAi rice (Figure 8d), resulting in enhanced salt stress tolerance. This result is in agreement with a report of increased salt sensitivity in *hkt1* knockout rice [45].

**Figure 9:**
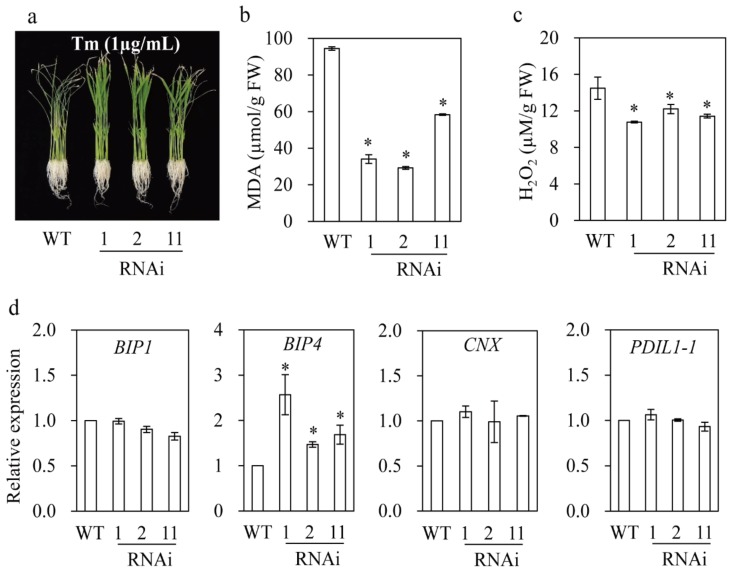
Tunicamycin tolerance in *M2H* RNAi rice. (**a**) Representative phenotypes of wild type and *M2H* RNAi rice in response to Tm. (**b**) MDA level. (**c**) Hydrogen peroxide level. (**d**) Relative expression levels of various chaperone-related genes. One-week-old rice seedlings were treated with 1 μg/mL tunicamycin (Tm) for three days under continuous light at 28 °C and recovered for two days in the absence of Tm under a 12/12 h light/dark cycle at 28 °C.

The *M2H* RNAi rice also showed enhanced tunicamycin (Tm) tolerance, as indicated by lower MDA and H_2_O_2_ concentrations than the wild type (Figure 9). Tm induces endoplasmic reticulum (ER) stress, and melatonin induces ER stress tolerance by ameliorating ER structure and enhancing protein folding by inducing the expression of a number of chaperone genes in *Arabidopsis* [22]. The *M2H* RNAi rice did not show a significant increase in the expression of chaperone genes such as *BIP1* (binding protein), *CNX* (calnexin), and *PDIL1–1* (protein disulfide isomerase-like; [46]) whereas the expression of *BIP4* was increased (albeit to varying degrees), suggesting involvement of chaperone genes in melatonin-mediated ER stress tolerance. In sum, the increased melatonin concentration due to inhibition of 2OHM conversion plays an important role in the tolerance of rice to various stresses.

## 4. Discussion

### 4.1. Distribution of Homologs of Genes Related to Melatonin, 2OHM, and c3OHM Biosynthesis during the Evolution of Land Plants

We assessed the phylogenetic lineages of genes related to melatonin, 2OHM, and c3OHM synthesis using the non-redundant protein sequence database [35,42]. Orthologs of *SNAT*, a pivotal enzyme in melatonin synthesis, are present from cyanobacteria to land plants whereas orthologs of *M2H* are absent in cyanobacteria and aquatic plants but present in land plants [35]. The *M3H* orthologs are only present in land plants. These data suggest that only land plants are capable of synthesizing 2OHM and c3OHM, whereas all organisms can produce melatonin. The emergence of *M2H* genes of the 2-oxoglutarate-dependent dioxygenase (2-ODD) superfamily matches the diversification of this superfamily during the evolution of land plants [47]. This implies that 2OHM played a role in the evolution of land plants from aquatic plants; unlike the latter, the former is exposed to cold, drought, ultraviolet (UV) light, and gravity. In response to these stresses, plants produce various metabolites; e.g., flavonoids and lignin in response to UV light and gravity, respectively [48]. By contrast, our results showed that a decrease in the 2OHM concentration confers tolerance to senescence, cadmium, salt, and tunicamycin (Figure 5, Figure 6, Figure 7 and Figure 8). This may be explained by the increased melatonin concentration due to inhibition of melatonin catabolism rather than by the decreased 2OHM concentration because melatonin is involved in the response of plants to abiotic stresses [18].

However, this link between an increased melatonin concentration and *M2H* suppression does not explain the reduced seedling growth and germination rate in the *M2H* RNAi rice (Figure 4), because melatonin promotes seed germination and seedling growth [17,43]. Thus, the observed phenotypes with and without stress conditions are to be decoded by the dual role of *M2H* expression. In fact, the melatonin concentration was lower in the *M2H* RNAi rice than in the wild type in the absence of stress (Figure 5a). In contrast, the suppression of *M2H* expression leads to a transient increase in the melatonin concentration upon stress, resulting in activation of other melatonin degradation pathways, such as the *M3H* pathway. The failure of shoot organogenesis from transgenic calli in the presence of *M2H* overexpression suggests that an elevated 2OHM concentration impairs rice shoot organogenesis by disturbing melatonin homeostasis or metabolism [34,49], or the melatonin-mediated balance between auxin and cytokinin, key hormones for somatic organogenesis [50]. In fact, exogenous 2OHM induces apoptosis in human cells [49] and enhances proline synthesis in plants [34], suggestive of biological roles different from those of melatonin.

The increased melatonin concentration in *M3H* RNAi rice under stress conditions was also due to inhibition of its conversion into c3OHM, but the *M3H* RNAi rice plants did not show the stress tolerance response compared to the wild type [42]. M2H is localized in chloroplasts and M3H in the cytoplasm [51]. Thus, suppression of *M2H* and *M3H* expression leads to an increase in the melatonin concentration in chloroplasts and the cytoplasm, respectively. The abiotic stress tolerance conferred by impaired melatonin catabolism is evidenced by an increased melatonin concentration in chloroplasts, but not in the cytoplasm. Differential melatonin concentrations in subcellular organelles has also been reported in rice *O*-methyltransferase (OMT)-knockout plants, in which a decreased melatonin concentration in chloroplasts resulted in premature leaf senescence [52]. This phenomenon has not been observed in a number of melatonin-deficient rice plants [10,18]. Therefore, the pleiotropic functions of melatonin in plant cells may be ascribed to its differential concentrations among subcellular organelles, similar to in animal cells [53]. Given the possible key roles of melatonin in different organs, it would be of interest to examine the phenotypic and physiological effects of targeted overproduction of melatonin in mitochondria, the endoplasmic reticulum, and the nucleus [54,55].

### 4.2. Functions of Diurnal 2OHM Production in Plants

Compared to other melatonin metabolites, such as 6-hydroxymelatonin, c3OHM, and 4-hydroxymelatonin, 2OHM does not possess potent antioxidant activity because its reactions with peroxy radicals are endergonic [56]. Thus, it likely has a different function from melatonin and other melatonin metabolites (such as c3OHM) in view of its free radical scavenging activity [42]. Nevertheless, the 2OHM and c3OHM concentrations exhibited a diurnal rhythm with a peak at night and the melatonin concentration peaked during the day in *Arabidopsis* [57] because high intensity light induces melatonin synthesis [23,58]. Thus, the increased 2OHM and c3OHM concentrations may be responsible for the decreased melatonin concentration at night in plants. In plants, the ROS and glutathione concentrations exhibit a diurnal pattern with a peak during the day [59]. This simultaneous induction of glutathione and ROS during the day modulates the cellular ROS levels. Such a diurnal rhythm of ROS concentration accelerates leaf senescence in *Arabidopsis* [60]. By contrast, 2OHM deficient rice plants were resistant to many abiotic stresses, indicating that 2OHM does not function in with ROS scavenging.

The melatonin concentration also shows a diurnal rhythm in *Arabidopsis* [57] possibly due to enhanced melatonin catabolism during the night. The day peak in melatonin concentration suggests a function as an antioxidant and as a signaling molecule by binding the melatonin receptor Cand2 to activate the mitogen-activated protein kinase (MAPK) cascade. In turn, this induces the expression of a number of downstream genes related to the innate immune response [33], unfolded protein response [22] and BR biosynthesis [10]. The daytime increase in the melatonin concentration likely enhances the ability of plants to adapt to adverse environments because several resistance mechanisms are light-dependent [61,62]. Thus, *M2H* plays an important role in regulating the melatonin concentration during the daily light/dark cycle. Therefore, suppression of *M2H* plays an important role in orchestrating melatonin levels by upregulating the expression of key melatonin biosynthetic genes such as *TDC*, *T5H*, and *ASMT* (Figure 5). Surprisingly, the poor drainage of 2OHM due to downregulation of *M2H* triggers an increased flux into c3OHM synthesis, resulting in a decreased melatonin concentration under normal conditions. By contrast, this anabolic and catabolic regulation among melatonin, 2OHM, and c3OHM is annihilated in the presence of abiotic stress; e.g., cadmium. Such abiotic stresses enhance accumulation of melatonin by inhibiting its conversion into 2OHM, which promotes resistance to abiotic stresses. Of note, 2OHM predominantly undergoes a tautomeric conversion into 2-acetamidolethyl-5-methoxyindolin-2-one (AMIO) which is more inert than melatonin [63]. Thus, it is recommended to avoid the name 2OHM, but it is easier to name 2OHM rather than AMIO in terms of melatonin catabolites in plants and animals.

## 5. Conclusions

In conclusion, we investigated for the first time the physiological roles of endogenous 2OHM in rice plants by using the molecular genetic strategy. The *M2H* RNAi rice seedlings produced lower 2OHM as well as melatonin in normal growth condition. Melatonin decrease in the *M2H* RNAi rice was ascribed to the increased synthesis of 3OHM via a detour catabolic pathway. Thus, the *M2H* RNAi rice exhibited a melatonin-deficiency phenotype such as a reduced germination rate and retarded growth. By contrast, this catabolic flux regulation was abolished upon challenge with cadmium stress. The *M2H* RNAi rice produced more melatonin than the wild type, leading to tolerance of cadmium, salt, senescence, and tunicamycin stresses. Therefore, *M2H* controls the melatonin level in plants under normal and stressed conditions. 2OHM rather than c3OHM is functionally linked to the physiological role of melatonin in plants by regulating its synthesis during normal plant growth and in the presence of stresses. The function of the diurnal rhythm in 2OHM concentration in plants warrants further study.
